# The complete chloroplast genome sequence of *Cornus Alba* L. (Cornaceae)

**DOI:** 10.1080/23802359.2021.1938727

**Published:** 2021-06-14

**Authors:** Wangjun Yuan, Sheqi He, Suping Zhang, Dongwen Chang, Yanxia He

**Affiliations:** a School of Pharmacy, Henan University, Kaifeng, China; b School of Life Sciences, Henan University, Kaifeng, China

**Keywords:** Chloroplast genome, *Cornus alba*, phylogenetic tree

## Abstract

*Cornus alba* has been used as antiphlogistic, hemostatic and diuretic treatments in Korea, and which is one of the most important ornamental shrubs in China. Here, we reported the complete chloroplast genome sequence of *C. alba*. The size of the chloroplast genome is 158,451 bp in length, including a large single copy region (LSC) of 87,778 bp, a small single copy region (SSC) of 18,927 bp, and a pair of inverted repeated regions of 25,873 bp. The *C. alba* chloroplast genome encodes 132 genes, including 85 protein coding genes, 38 tRNA genes, and 8 rRNA genes. Phylogenetic tree showed that *C. alba* with the species of *C. macrophylla* and *C. sanguinea* formed a strongly supported clade.

The genus *Cornus* Linnaeus (Cornaceae) consists of more than 55 species, mostly shrubs and small trees (Bjorøy et al. 2007). *Cornus alba* Linn., also known as red-barked or Siberian dogwood, is native to Siberia, northern China and Korea (Park et al. 2016). The stems and leaves of *C. alba* have been used as antiphlogistic, hemostatic and diuretic treatments in Korea. This plant is one of the most important ornamental shrub in China due to its colorful autumn leaves, its crimson bark in the winter season, white flowers and its berries with a bluish white metallic shine (Luo 2020). The advent of high-throughput sequencing technology made it possible to obtain large amounts of genomic data rapidly (He, Yuan, et al. 2017). In this study, we reported the complete chloroplast (cp) genome of *C. alba* (Genbank accession no. MW368898) to provide genomic and genetic information for further research.

The fresh leaves of *C. alba* were collected from the campus of Henan University, China (34°49'19.68"N, 114°18'51.32"E), and a voucher specimen was kept at the Herbarium of Henan University (Voucher number: Wangjun Yuan 2020080601, Kaifeng, Henan Province, China, Wangjun Yuan and 10200068@vip.henu.edu.cn). Total genomic DNA was extracted with the modified SDS method. Sequencing libraries were generated using NEBNext® Ultra™ DNA Library Prep Kit for Illumina (NEB, Bloomington, IN) following manufacturer’s recommendations, which were sequenced using Illumina NovaSeq PE150 at the Beijing Novogene Bioinformatics Technology Co., Ltd. (Beijing, China). Approximately 5 Gb raw data were generated, and these were trimmed and assembled into contigs using a CLC Genomics Workbench 9.5.2 (CLC Inc., Aarhus, Denmark) with the chloroplast genome sequence of *C. macrophylla* (GenBank accession no. NC044810) as a reference. The complete cp genome was assembled and annotated using Geneious R11 (Biomatters, Auckland, New Zealand) following description (He, Liu, et al. 2017; Liu et al. 2018).

The chloroplast genome of *C. alba* was 158,451 bp in length, containing a large single copy region (LSC) of 87,778 bp, a small single copy region (SSC) of 18,927 bp, and a pair of inverted repeat (IR) regions of 25,873 bp. Genome annotation predicted 132 genes, including 85 protein coding genes, 38 tRNA genes, and 8 rRNA genes. The overall GC-content of the chloroplast genome was 37.9%, and the corresponding values in the LSC, SSC, and IR regions were 36.0%, 32.2%, and 43.1%, respectively.

The phylogenetic tree including *C. alba* and other 18 species was constructed by whole cp genome sequences using the maximum-likelihood(ML) method implemented in RAxML-HPC v8.1.11 on the CIPRES cluster , which showed that *C. alba* with the species of *C. macrophylla* and *C. sanguinea* formed a clade with a 100% bootstrap value (Figure 1). The topology of Cornales is identical to that of Xiang et al. (2011).

**Figure 1.**
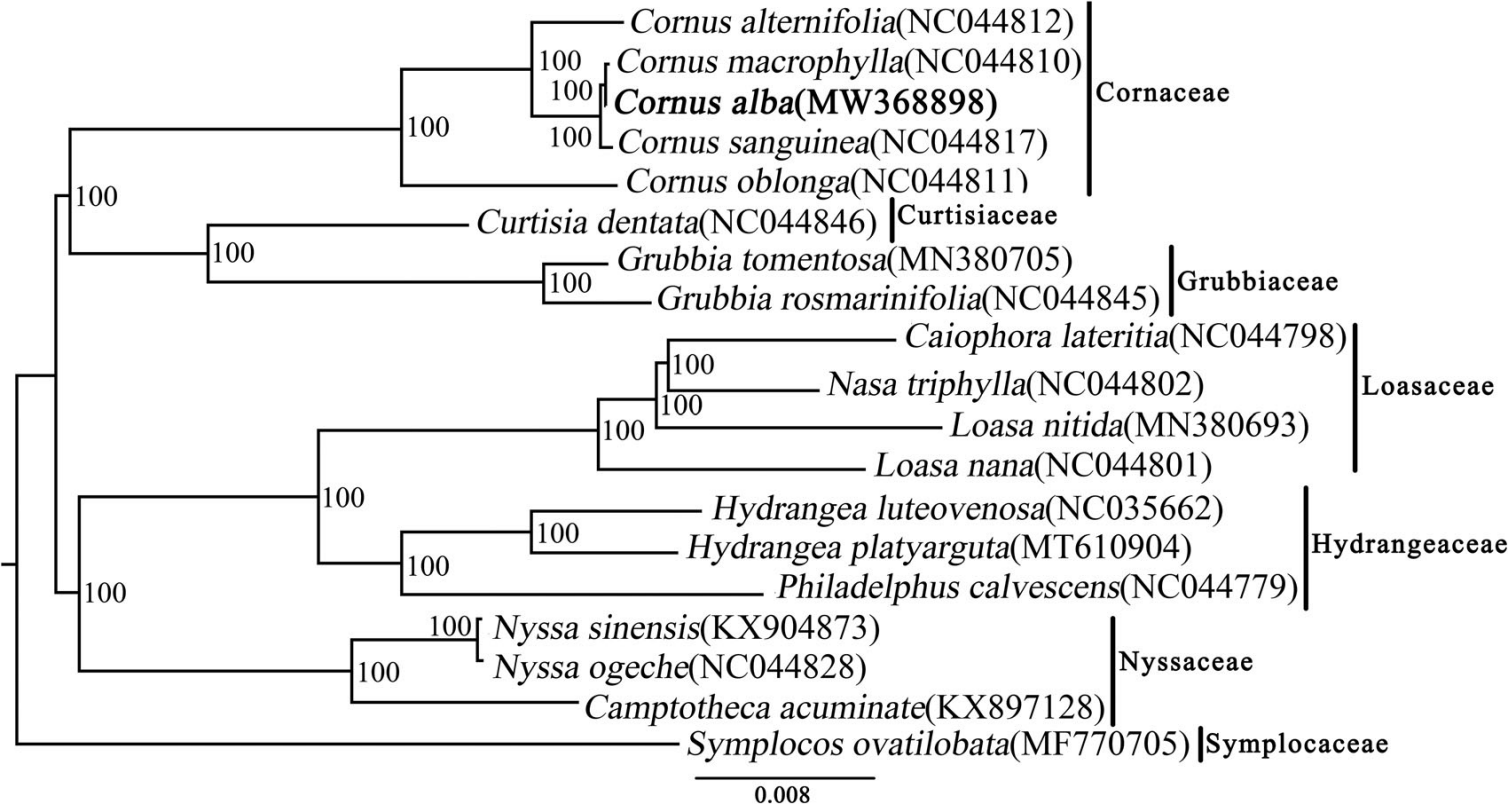
Phylogenetic tree inferred by the maximum-likelihood (ML) method based on 19 representative species.

## Data Availability

The genome sequence data that support the findings of this study are openly available in GenBank of NCBI at https://www.ncbi.nlm.nih.gov/ under the accession no. MW368898. The associated ﹡BioProject﹡, ﹡SRA﹡, and﹡Bio-Sample﹡ numbers of the raw sequence data and the genome are PRJNA720529, SRR14181230, and SAMN18653152, respectively.

## References

[CIT0001] Bjorøy Ø, Fossen T, Andersen ØM. 2007. Anthocyanin 3-galactosides from *Cornus alba* 'Sibirica’ with glucosidation of the B-ring. Phytochemistry. 68(5):640–645. 17207823 10.1016/j.phytochem.2006.11.028

[CIT0002] He Y, Yuan W, Dong M, Han Y, Shang F. 2017. The first genetic map in sweet osmanthus (*Osmanthus fragrans* lour.) using specific locus amplified fragment sequencing. Front Plant Sci. 8:1621. 29018460 10.3389/fpls.2017.01621 PMC5614988

[CIT0003] He YX, Liu LX, Yang SH, Dong MF, Yuan WJ, Shang FD. 2017. Characterization of the complete chloroplast genome of Chinese fringetree (*Chionanthus retusus).* Conserv Genet Resour. 9(3):1–4.

[CIT0004] Liu LX, Li P, Zhang HW, Worth JRP. 2018. Whole chloroplast genome sequences of the *Japanese hemlocks*, *Tsuga diversifolia* and *T. sieboldii*, and development of chloroplast microsatellite markers applicable to East Asian Tsuga. J Forest Res. 23(5):318–323.

[CIT0005] Luo QQ. 2020. Molecular identification of collect otrichum species causing anthracnose disease of *Cornus alba* “Bud′s Yellow. Plant Protect Chin. 46(5):102–109.

[CIT0006] Park KH, Yin J, Yoon KH, Hwang YJ, Min WL. 2016. Antiproliferative effects of new dimeric ellagitannin from *Cornus alba* in prostate cancer cells including apoptosis-related S-phase arrest. Molecules. 21(2):137. 26805810 10.3390/molecules21020137 PMC6273526

[CIT0007] Xiang QYJ, Thomas DT, Xiang QP. 2011. Resolving and dating the phylogeny of Cornales – effects of taxon sampling, data partitions, and fossil calibrations. Mol Phylogenet Evol. 59(1):123–138. 21300164 10.1016/j.ympev.2011.01.016

